# Transactions of the Medical Society of the County of Kings

**Published:** 1865-02

**Authors:** 


BUFFALO

Medical and Surgical Journal.

VOL. IV. FEBRUARY, 1865. No. 7.

ART. I.--*Transactions of the Medical Society of the County of Kings.*

REGULAR MEETING, JANUARY, 1863.

*Rupture of the Uterus.*

DR. E. N. CHAPMAN reported the following cases:

Was called on the morning of January 26th, to visit Mrs. R. in consultation with Dr. Leach. Case of difficult labor. She had borne several children, but previous labors had been tedious. Her health had been good, was accustomed to labor, was strong and masculine in appearance. She had been in labor from 4 o'clock A. M. of the 22d, and was attended during this time by a midwife, until the evening of the 25th, when Dr. L. was called in. Until some time on the 24th, the pains were represented as strong and regular, and occurring at intervals of five minutes. During the day (24th) they were forcible and almost continuous, and yet the child was not moved from its position on the morning of 24th, as represented by the midwife. Dr. L. found the head firmly impacted in the pelvis, so firmly that he could not move it in any way. The pains were constant, high up, and did not seem to possess expulsive force so far as any effect upon the child was manifested, but seemed of a circular character. Ergot was administered and an attempt was made to apply the forceps, but failing to obtain satisfactory results, the Doctor left her at 2 A. M. believing that craniotomy must be performed, and requesting a consultation at 9 A. M. I saw her at about half past 9. Upon the Doctor's making an examination he remarked that the case was worse than he had represented, as the head could not be reached by digital examination. The patient complained of considerable distress in the region of the umbilicus—pulse rapid and feeble, extremities cold, countenance anxious, and she requesting help. She had considerable hemorrhage during the night of the 25th. Upon introducing the hand the head was found lying entirely above the brim of the pelvis, and movable. Rupture of the uterus was diagnosed, and notwithstanding her extreme exhaustion, it was deemed advisable to attempt to turn and deliver, as affording the best prospect in the case. Accordingly the feet were found and brought down, and the body brought forward without any unusual difficulty. There was no contraction of the uterus upon the introduction of the hand, nor from the exhibition of ergot. Nor, although stimulants were freely administered, did much reaction take place. Upon attempting to bring down the head, we were foiled. The position of the child was with the face toward the sacrum of the mother. There being no evidence of life in the fœtus, either in the pulsation of the heart or cord, forcible traction was made both by the body alone, and by the body and under jaw. While making traction the spinal column gave way. The forceps were then applied, but with no better result, and we resolved to break down the head and deliver by piece-meal, as it was evident there existed too great a disproportion between the head and pelvis to allow of delivery in any other way, as the head had become almost detached from the body. The head was pierced and the bones somewhat broken down, when the sinking of our patient warned us to desist and place her upon the bed more fully, when she soon after expired, leaving us a headless trunk, and she undelivered.

As to the amount of force employed in thus decapitating this fœtus, there exists a little difference of opinion, and but a little. I believing that Dr. L. made slight traction with one hand, in addition to that which I was making at the time the spinal column gave way. The Doctor is confident that he did not. In any event the extra power was but a few pounds, and no more force was applied than I have frequently applied in similar cases, but without a like result. The fœtus appeared healthy, with no evidence of decay. The head was unusually large, but not hydrocephalic, as no water issued from the puncture. The pelvis was considerably contracted in its autero-posterior diameter, at the brim. No post mortem could be obtained.

On the 5th of February I was called in consultation with Doctors Rottou and White to see a case so like the above that I report it in connection.

Mrs. E——, a lady of 27 years of age, under medium size, in good health, was taken in labor with her fourth child the day previous. Dr. R. was called in the afternoon, and finding no necessity for remaining, returned home, and was recalled about midnight—labor was progressing well, head presentation, pains not severe until 3 or 4 o'clock A. M. when they became very frequent, and very strong, so much so as to create some uneasiness in his mind in regard to the patient's safety. The abdomen was quite prominent, and the integuments seemed somewhat attenuated. Although the pains were very strong the head did not advance, and up to 9 o'clock A. M., after six hours of strong pains it had just begun to enter the brim of the pelvis. At this time, the Doctor, having his open palm upon the abdomen to give some gentle support felt the uterus give way on the left side, about three or four inches above the pubis, and what seemed to be a knee thrust itself through. The patient at once understood the nature and consequence of the disaster, and so expressed herself. Dr. R. assisted by Dr. White, who was at the time present, at once proceeded to deliver her by version. The feet were found and brought down, and the body of the child delivered without trouble—persevering efforts were made to complete the delivery, both by traction upon the body, and also with the fingers in the mouth to assist, and also to guide and rotate the head; also by the application of the forceps, but after persevering and fruitless efforts, delivery in this way was abandoned and craniotomy resolved upon unless the forceps could be more successfully used with the body removed so as to give more freedom of action. With this view the fœtus was decapitated. It was at this stage of labor that I first saw her, and attempted to apply the forceps to the head, lying entirely above the brim of the pelvis and within the cavity of the uterus. The head could not be steadied so as to apply the forceps, and embryotomy was decided upon as a last resort. The lady opposing this procedure, and her spiritual adviser having just arrived, when, after some delay, upon the departure of the priest, her pulse was found to be 170 and weak, and everything indicating speedy dissolution. Further proceedings were deferred until reaction should take place. Stimulants, beef tea, and anodynes were administered, and although her sufferings were great, and hemorrhage continuous from the time of the rupture, yet during the night she so far rallied as at 5 o'clock A. M, to feel able to endure the removal of the head, and we were again summoned for that purpose, but only to find her moribund, something less than twenty-four hours after the rupture. No post mortem could be obtained.

### *Cerebral Apoplexy.*

Dr. Speir presented a specimen of cerebral apoplexy, produced by the rupture of the *transverse branch of the basilar artery.*

A man, aged 50, fell down a flight of stairs, was taken up *comatose*, and brought to the City Hospital, where he died in an hour after admission. On removing the calvarium, considerable blood exuded; and on opening the duramater, there was a clot of not less than five or six ounces, compressing the brain to two thirds of its volume. The *liver* was fatty; the *kidneys* granular, the *capsules* being removed with difficulty. In the *arteries*, generally, there was extensive *atheroma*, particularly in the brain.

### *Scirrhus of the Rectum and Uterus.*

Dr. Speir also presented a specimen of *scirrhus of the rectum and uterus*, producing *stricture* of the *rectum*. (The case belonged to Dr. H. S. Smith.) The uterus and rectum were united by a scirrhus mass, dislocating the uterus backwards and to the right, and producing a stricture of the rectum, through which the first finger could with difficulty be passsed. The portion of gut above the stricture had formed a *pouch*, and was continuous with the lower portion and *anus*, by a tortuous passage through the stricture and behind the body of the uterus, giving to the canal the form of an inverted S, the stricture being in the middle, thus ∽.

Dr. Bell recurred to the first case presented by Dr. Spier. He saw the patient soon after he fell down stairs, and finding symptoms of compression, had him removed to the hospital. Dr. Bell raised the question, as to whether the *fall* had ruptured the artery, or whether the *previous rupture* of the badly diseased artery and *apoplexy* had caused the fall? The man, though of intemperate habits, was said to have been sober at the time of the accident, and carefully feeling his way through a dark stair-way, (it being about 8 o'clock in the evening.)

Dr. Hutchison regarded the question, raised by Dr. Bell, an interesting one, bearing upon *traumatic apoplexy*, without *fracture*, which was exceedingly rare.

Dr. Enos inclined to the opinion that the artery was ruptured by the fall.

Dr. Hart was familiar with the post mortem appearances in apoplexy, but in no case had he observed a coagulum, which he thought suggestive in this case, that the apoplexy may have preceded the fall.

Dr. North stated that he had seen a case, examined only a few hours after death, in which the whole cerebal surface was covered with a *clot*.

Dr. Speir had seen several cases of apoplexy *in* the cerebral substance, always coagulated; but he had not, ere this, seen one with *a clot on the surface*.

### *Absence of the Epiglottis—Ulceration of the Larynx.*

Reported by Dr. D. C. Enos.

Mrs. M., aged 33, had enjoyed good health till about three years since, when she was taken with inflammation about the fauces and larynx; she was attended by Dr. Batcheldor, of New York, who applied a solution of nitrate of silver. Dr. B. attended her till within two weeks of her death, when I was called to see her. She was then weak, but still able to sit up; she could only speak in a whisper—she lost her voice two years ago; her respiration was very difficult—both inspiration and expiration prolonged, labored and noisy; she had slight cough, and was weak and nervous. There was no enlargement of the larynx externally, neither was it tender, on pressure. The stridulous respiration prevented a satisfactory examination of the chest; an asthmatic wheezing seemed to pervade the lungs throughout; they were everywhere resonant on percussion. The remedies used were tonics and nervines. On the day of her death Dr. Batcheldor saw her with me; this was at two o'clock. She seemed failing, but did not excite apprehension of a speedily fatal result. The breathing was not more difficult than it had been, although she coughed and raised much more—from recent bronchial inflammation. She died in the evening of of the same day.

There had been no sudden alteration in the condition of the larynx, but the large quantity of mucus in the air passages, superadded to the diseased and contracted larynx, caused her sudden death. The post mortem examination showed that the larynx internally was much diseased. The epiglottis was entirely absent, from ulceration, and there was an irregularly hardened and knobbed cicatrix in its place at the end of the hyo-epiglottic ligament; and the same uneven structure existed in the place of the arytenoid epiglottidean folds which were effaced. The same diseased condition also extended into the ventricles and laryngeal pouches. The true vocal cords and the arytenoid cartilages were also so changed by the disease as to materially diminish the area of the rima glottidis. The trachea and bronchial mucous membrane were acutely inflamed. She had no difficulty in swallowing either liquid or solid food. This case, therefore, illustrates the physiological fact that the epiglottis is not necessary to prevent food from entering the larynx in the act of deglutition. It is well known that birds have no epiglottis, and that dogs and other animals, from which the epiglottis has been removed, find no trouble in swallowing. The inferior constrictor muscle of the phaxynx, inserted as it is into the posterior part of the thyroid cartilage on either side, compresses these sides together as the larynx rises in deglutition, in such a manner as to effectually close the chink of the glottis by bringing the vocal cords in contact. This muscle, therefore, at every contraction, performs two important offices, that of keeping food from entering the trachea and at the same time urging it onward towards the stomach.

---

### REGULAR MEETING, MARCH, 1863.

### *Black Vomit, with Discussion as to its Etiology.*

Dr. J. T. Conkling reported the following case:

A seaman, aged 19, left New Brunswick in October last for Kingston, Jamaica. While there, he had, he said, bilious fever, and was confined to the hospital several weeks. He remained on the island two weeks longer in a boarding-house, where his food consisted only of bread and salt fish. On the first of February he left in the barque John Bolton for Philadelphia, and, though very feeble, worked his passage during a voyage of eighteen days. From Philadelphia he came directly to this city, arriving here on Thursday morning, Feb. 19th. He partook of a breakfast of beef-steak and coffee, and at dinner, of corned beef and cabbage. In the evening he vomited, and had a restless night. His sister said she gave him on Friday, salts and cream of tartar, which produced vomiting and purging. Friday evening, at seven o'clock, he commenced vomiting large quantities of dark fluid matter. I first saw him on Saturday morning, at three o'clock. He was suffering with severe pains in the bowels; had intense thirst; would drink a few swallows of water, and immediately vomit a pint or more of black fluid, which excoriated his throat and had a very acid odor; his pulse was natural; eye clear; and skin normal in color and temperature. His bladder was empty, and he told me he had passed urine the evening before. The vomiting continued during the day and following night. His mind remained clear. There was a constant restlessness and shifting of position in search of ease. There was no evacuation of urine or fæces after I saw him. His strength failed gradually, and he died at nine o'clock on Sunday morning, thirty-eight hours after the first occurrence of the dark vomited matter.

Drs. Enos and Bell saw him previous to death. The post-mortem and microscopical examinations were made by Dr. Speir. The following is Dr. Speir's report:

*Autopsy, seven hours after death.*—Body well formed but emaciated; not yet cold; rigor mortis not well pronounced; surface tinged livid; frothy mucus filling the nostrils. The pectoral and abdominal muscles were of a very dark color, almost purple. *Thorax.*—Old adhesions at the posterior part of the left lung. Intense sanguineous engorgement of both lungs. Incisions made in any part of the lungs gave rise to an abundant flow of very dark-colored blood. The pericardium contained about an ounce of yellow serum. The heart was full sized, firm, usual amount of fat; veins upon the surface distended with blood; the cut surface presented a darker hue than usual; intensely black clots in right ventricle and auricle; a very small light-colored clot in right ventricle, and a very small black clot in the left auricle; slight thickening of semilunar valves of aorta; otherwise valves perfect, *Abdomen.*—Liver very dark, between a purple and chocolate color; appeared normal in size; borders sharp and well defined; a few patches upon the anterior surface of a lighter color than the rest of the organ, having a slight yellow tint. The organ was firm; its cut surface was smooth, and, contrary to opinion based upon its dark color, it was dry, little or no blood making its appearance in the course of the knife. Its ducts were stained with yellow bile. *Stomach.*—Very large, extending down to umbilicus and into right lumbar region, and filled with two quarts of black fluid similar to that vomited by the patient; very acid odor; mucous membrane near the pylorus much congested and bared of mucus; the other portions were covered with thick mucus mixed with dirty black material like the vomita. *Duodenum.*—Filled with a similar black fluid; mucous membrane dark colored; not congested; intestines at some points much congested; they contained but little fæcal matter, of a yellow color, with only slight odor. Peyer's patches were healthy. The *spleen* was twice its normal size, of a dirty black color, and becoming diffluent. The *kidneys* were congested, nearly normal in size, and firm; their capsules firmly attached; cut surface smooth, dark colored, and soon covered by a layer of dark blood. The *supra renal capsules* were of a dark color, their cortical and medullary substance not being well defined. The head of the *pancreas* was a little enlarged, otherwise normal, The *bladder* contained about four ounces of reddish urine. The *blood-vessels* contained but little blood, and that of dark color.

*Microscopical and Chemical Examination,*—The *black vomit* was very acid. Seen through the microscope with a power of about one-fourth of an inch, it was composed of a thin, transparent fluid, floating in which were found—striated muscular fibres (ingesta), altered starch corpuscles, a few oil globules, altered blood corpuscles, and numerous sarcinæ ventriculi, the whole field being covered with black amorphus granules, varying in size, and some of them aggregated together; many of them, on changing the illumination and focus, transmitted a deep red light. They were not acted upon when treated with acetic acid, but were dissolved by ammonia and liquor potassæ, giving a deep red color. They were considered to be granules of hœmatoidin. *Liver.*—Its cells were normal, with the usual amount of fat. A few cells were found in process of fatty degeneration; these were rare. *Amorphous granules*, similar to those found in the black vomit, were everywhere present, but less abundant than in the vomita. In some places the granules were fine, and gave to the field a yellow tint. A few crystals were found, probably so-called blood-crystals. *Heart.*—Muscular fibres perfect; granules of hæmatoidin present, but not abundant. *Kidneys.*—A few of the tubes were partially denuded of their epithelium; abundant granules of hæmatoidin; otherwise healthy. *Spleen.*—Considerably disintegrated; abundant granules of hæmatoidin; blood corpuscles much changed and broken down, being swollen, oblong, irregular, and granular. *Pancreas* healthy; contained a very few granules of hæmatoidin. *Supra-renal capsules* apparently healthy; granules of hæmatoidin more abundant than in pancreas. *Urine* albuminous; scales of epithelium from bladder and ureters; urate of ammonia, blood corpuscles, and granules of hæmatoidin.

The dark coloration of the tissues seemed to be due to the abundant granules of hæmatoidin, the shade of color depending upon the size, depth of color, and aggregation of the granules, the very fine ones giving a yellowish tint to the field of the microscope.

As the coloring matter of the bile (cholephyrrhine) is closely allied to hæmatoidin, it would seem possible for the latter, under certain circumstances, to give a yellow color to the skin and tissues, similar to that sometimes produced by the coloring matter of the bile, instead of the purplish color observed in this case.

REMARKS BY DR. A. N. BELL.

Dr. Bell stated that, besides having seen the case of fever reported by Dr. Conkling, he had also been privileged to assist in the post-mortem, and that, notwithstanding the unusual circumstances of climate and season, he thought the evidence conclusive that it was an uncomplicated case of yellow fever. The yellowness of the skin and the fawn color of the liver, which were absent in this case, though generally present in yellow fever, were by no means essential in the diagnosis, especially when we have, as in the case reported, the most pathognomonic of all the symptoms, *black vomit*, and the most pathognomonic of all the post-mortem appearances, *fatty degeneration of the liver*. And, in addition to these conditions, we also have a large quantity of "black vomit" in the stomach and intestines; a fluid blood, with broken-down corpuscles; an emptiness of the blood vessels; an intensely congested and almost diffluent spleen—indeed, *all* of the most usual conditions of a rapidly fatal case of yellow fever. Dr. Bell also stated, in connexion with this case, that he had observed, in common with others who had had much experience in yellow fever in different climates, that it usually ran a more rapid course, and was more fatal in climates where it rarely occurred than where it is indigenous. He accounted for this by analogy: in that the organism of individuals attacked by it under such circumstances was deprived of the benefit of a gradual adjustment to the influence of the poison, on the same principle as that a vigorous bird speedily perishes in an atmosphere which will sustain one gradually brought under its influence for a much longer time. In such cases, too, we should expect to find a less perfect symptomatology and a less perfect degree of pathological lesion, as in the case under discussion. Yellowness of the skin, though a common symptom, is nevertheless frequently altogether absent. It rarely occurs in any case before the third day, on the decline of febrile excitement, and is usually regarded as the second stage of the disease. Hence, in those cases which are fatal during the period of excitement, yellowness of the skin is commonly absent, or does not occur until after death. Fatty degeneration of the liver, also, in the case reported, seems to have but just commenced; and the liver, instead of being fawn color, as it probably would have been had the case been of longer duration, was just beginning to be so in spots. Whether fatty degeneration of the heart is consequent upon a still more protracted continuance of the disease, cannot as yet be made apparent. Prof. Riddell, of New Orleans, regards "molecular degeneration of the heart" as being a much more constant lesion than fatty degeneration of the liver. According to his (Dr. Bell's) experience the converse is the case. Of four cases examined microscopically last summer, in the Floating Hospital, they all had fatty degeneration of the liver, and one only molecular degeneration of the heart; that one died on the seventh day. Fatty degeneration of the heart is a comparatively recent discovery in yellow fever,* and *by the microscope only*, while the commonly present fawn-colored liver in this disease has for a long time *been supposed* to indicate a fatty degeneration of that organ. Pathologists of the present generation will hardly accept the proof of fatty liver on the mere presence of yellowness; or, on the other hand, regard the absence of this color as corresponding with the absence of this important lesion. The now universally acknowledged importance of the microscope applies to nothing with more force than to yellow fever, and, while it had been his opportunity to have passed through several epidemics of yellow fever, and to have made a large number of post-mortem examinations in this disease, its true lesions he had never, until recently, observed. It was his firm belief that the thorough and accurate delineation of this single case by Dr. Speir is of more value to the profession than all the experiences of a Chisholm. In conclusion, Dr. Bell read the following extract from a letter that he had received from Dr. La Roche, in reply to one that he had written to that distinguished authority regarding the history of this case:—

"The *John Bolton* entered on her voyage to Philadelphia on the nineteenth of January, at Porto Cabello, and, touching at Kingston, Jamaica, left that port on the second of February. She arrived in Philadelphia on the eighteenth of February, after a passage of sixteen days, and entered on the nineteenth. The Captain reports no sickness either in Porto Cabello or at Kingston, and the vessel had no sickness on board from the commencement of the voyage, and the officers and crew were all well when they were examined. The vessel cannot have been in foul condition when she reached Philadelphia. Had there been anything wrong about her, the Port Physician, Dr. Trenchard, who is a careful officer, would not have failed to report the fact to the Board of Health. From all this it would appear, that the patient proceeded to Brooklyn on the very day of the visit of the Port Physician; that although he may have been ailing at the time of the examination, he was not sufficiently so to attract the attention of that officer; that he did not suffer from any poison generated or lurking in the vessel since no one else on board, during the voyage or after the arrival of the vessel, was affected in like manner, or in any way approaching to it. Hence the case, supposing it to have been one of yellow fever—and on this point I think, there can be no doubt, the absence of jaundice in so rapid an attack

* 1853. Riddell, Microscopical Obs. pertaining to Yellow Fever.

being a matter of not the smallest consequence—the case, I say, may very fairly be referred to a poisonous impregnation received at Porto Cabello, or perhaps more likely at Kingston. It is true, as the Captain reported, that 'no sickness prevailed in the harbor or on shore, or at either of the said places;' but it is well known that in tropical seaports the poison of yellow fever is never so effectually banished, however healthy the season may be considered,* as not to pounce upon some unlucky fellow peculiarly predisposed to its morbific influence. If I am not mistaken in what precedes, you have had before you an instance of rather long incubation—of thirty-one days—if the poison was received at Porto Cabello, and of nineteen days if the disease is to be traced to Kingston. I incline towards the latter view, inasmuch as we can hardly suppose that the poisoning took place on the very day of departure from either port, and a few days more would make the period of incubation from Porto Cabello too long, though such long periods, and even much longer ones, are not unknown, but they are comparatively rare."

REMARKS OF DR. D. C. ENOS.

Dr. E. said he saw the patient in consultation with Dr. Conkling. He thought the case was an obscure one, presenting some of the phenomena of the yellow fever, while many of the symptoms of that disease were altogether absent. The only prominent symptom of yellow fever was the black vomit. Doubtless, as Dr. Bell says, this is the most pathognomonic of all the symptoms of yellow fever; but it would be as erroneous to assert that every case of black vomit is one of yellow fever, as it would be to maintain that yellow fever could not occur without it. When speaking of black vomit, Dr. La Roche says, "considered by itself, without reference to other phenomena by which it is preceded and accompanied, and especially when noticed in a *single* or *a few spasmodic instances*, the black vomit is not sufficient to stamp the disease in which it occurs as being the true yellow fever. While, on the other hand, its occurrence in this disease is not sufficiently constant and necessary to justify us in refusing to recognise as such cases which present its other symptoms, merely on the ground that black matter has not been ejected from the stomach." His citations from the best authors to prove both these propositions are ample and conclusive, covering fifteen pages of his great work on yellow fever. Hence, if the case detailed be one of yellow fever, in order to establish it beyond a doubt,

* Yellow fever existed at both Porto Cabello and Kingston last summer. A. N. B

the antecedent history, concomitant symptoms, and post mortem lesions, must give the necessary corroborative evidence. What is their testimony? To determine this question properly, the points of the case which do not assert, as well as those which do, with the known phenomena presented by yellow fever, could be noted.

Some of the facts in this case which do not agree with those *most generally present* in yellow fever, are:

1st—*The period of incubation was longer.* The best authorities say the stage of incubation varies from a few hours to five or ten days—*occasionally, but very rarely*, to sixteen, twenty, or more days. In this case it was at least nineteen days, and doubtless more, for, as Dr. La Roche says, it is not probable he took the poison the very day he left. His history as given, though not incompatible with yellow fever, still offers no *presumptive evidence* in favor of such a theory, but rather the reverse, since this compatibility itself requires to be established by the most unequivocal evidence that the case was one of yellow fever, its symptoms and its lesions being incapable of any other interpretation. Such a case being made out the mind would be compelled to believe that the man passed in an uninfected ship, this, to say the least, unusually long period of incubation; that he and he alone took the disease at Kingston or Port Cabello, and at a time when it was not prevailing in either place; and in mid-winter he must have taken the malady from its slumbering dregs or from its nascent causes, which it is said "are never effectually banished from tropical seaports."

2d—*It was not ushered in with a chill.* "There is probably no disease," says Bartlett, "unless it is puerperal peritonitis, the access of which is more *invariably attended* by a chill or rigor than this."

3d—*Pain in the head was absent.* In yellow fever, according to the same authority, it is almost invariably present—"generally it is acute and violent." Mr. Pym says, "The most characteristic symptom of the disease is the peculiar pain in the forehead and eyeballs, with the drunken appearance of the eye."

4th—*The patient had severe pains in his bowels*, and not in his back, loins, and limbs, which are generally so constant and intense in yellow fever.

5th—*The color of the skin was normal.* "In fatal cases of yellow fever, yellowness of the surface is almost always present."

6th—*The eyes were not suffused, injected, or yellow*—symptoms, some or all of which usually occur in the course of fatal cases, at least, of the genuine typhus icterodes.

7th—*The patient had excessive thirst*, which Sir Gilbert Blane says is not *usually* the case in yellow fever—so say Bally, Jackson, Chisholm, Clark, and Dr. Lewis, of Mobile.

We have then the highest authority for believing that all these symptoms are *generally* present in yellow fever. Cases, however, do occur, in which one or more of them are absent, but rarely, if ever, in which all of them are.

These are some of the facts which, during the progress of the case, led Dr. E. to doubt that it was one of genuine yellow fever, notwithstanding the presence of the copious black vomit, which was so acid that it excoriated the fauces, and notwithstanding the constant uneasiness and jactitation of the patient, which are generally so characteristic of the disease. This doubt the post-mortem examination so carefully and so creditably made by Dr. Spier fails to remove.

The livid tinge of the surface which he describes does not equal "the yellow color varying from a pale or light to a dark orange or brown tint," which La Roche says is generally present:—"Sometimes it is greenish, mahogany, leaden, purple, or black." The lungs were engorged with black blood, as they sometimes are in yellow fever, though Drs. Physick and Cathrall (1793) found the lungs perfectly sound; so as a general thing did Harrison, La Roche, and others. Not unfrequently, however, the lower portions, of the lungs are engorged with altered blood. This is frequently so in other diseases characterized by blood dyscrasia. The *dark clot* found in the right cavities of the heart in this case is like that frequently found in yellow fever, though Bally and Pinnell found it of a light amber color. The dark clot is also found in other diseases. The stomach contained the same dark fluid as that vomited, so did the intestines.

Dr, Bell relies mainly on fatty degeneration of the liver as the "pathognomonic lesion" of yellow fever. This is unfortunate for his diagnosis in this case, for the liver was not fatty in the true sense of the term. Dr. E. said he examined the hepatic cells and tissue with Dr. Spier. The cells were unusually normal—less fatty than those of any liver examined at the B. C. Hospital, in the last six months. A few cells contained a little more than the normal quantity of fat, but this can be found in most livers and in various diseases. Hæmatoidin was found abundant in the hepatic tissue, which Dr. Clark says is not found in yellow fever. The liver had not the yellow color so much insisted on by Louis, as the *peculiar* condition in yellow fever, and which is now supposed by some to be owing to acute fatty degeneration. M. Catel says, of 150 cases of yellow fever, all the livers were abnormal in color—discolored, and yellow, fawn or drab. Dr. Spier says, this liver was very dark, between a purple and a chocolate, with a few patches on the anterior part a little lighter—slightly yellow in tint. The general and microscopic appearance of this liver, then, is unlike that usually seen in yellow fever. The *spleen* was *very soft;* whereas in yellow fever, says Bartlett, it is not the seat of any frequent and important alterations. Louis and Trousseau found it somewhat softened in about half the cases. Dr. Bache, in two out of ten. Dr. La Loche says it is often little, if at all, changed. The kidney was not degenerated, as in Bright's disease. The urine was scanty, though he passed it, he said, the evening before Dr. Conkling saw him. That found in the bladder was albuminous. It is scanty or suppressed in yellow fever; so it is in cholera, or in any disease in which the fluids are passing off rapidly by the skin, or, as in this case, by the gastro-intestinal mucous membrane. The albumen was due to the presence of blood in the urine, as proved by the microscopic examination. The kidneys were congested, but normal in *size* and structure. Dr. Pinnell says, in fatal cases of yellow fever the kidneys are in a condition like that witnessed in Bright's disease. Dr. Blair met with only a few cases of *blood urine* in yellow fever. He regarded it as a favorable symptom.

The muscular fibres of the heart were perfect. In yellow fever Prof. Riddell found them degenerated, all traces of striation having disappeared.

Dr. Bell said the "liver was just beginning to be dissolved," "that fatty degeneration had but just commenced." This may be so, but it hardly answers the purpose of a "pathognomonic" diagnosis. We cannot well say what it was about to do ; all we can say, in point of fact, is, the liver was *not* fawn-colored, it was *not* in a state of fatty degeneration. We might as well say the kidneys were in the same morbid condition, because, though otherwise sound and healthy, a few of the tubuli uriniferi were bereft of their epithelium. So far as the heart is concerned, there is no room to predict that any such process was about to take place, for the record is, its "structure was perfect." The molecular disarrangement of the cardiac muscular fibres, which Prof. Riddell found so constant in yellow fever, had *not even commenced.*

Hence, from all these facts, Dr. E. inclined to think the case was not one of *genuine yellow fever*. He expressed this doubt with some hesitation, on account of the positive opinion of Dr. Bell, coincided in, it seems, by the distinguished author of the letter he read. It is proper to remark, however, that Dr. La Roche did not see the patient during life, nor witness the examination of the body after death.

Dr. Bell's explanation of the imperfection of the symptoms and lesions in this case is its "fatal rapidity," which, he says, is usual in climates where yellow fever rarely occurs; "since," he remarks, "the organism is deprived of a gradual adjustment to the influence of the poison, on the same principle as that a vigorous bird speedily perishes in an atmosphere which will sustain one gradually brought under its influence for a much longer time. But surely we must believe that any bird, whether slowly or suddenly brought into a poisonous or foul atmosphere, will live longer or die more gradually when *totally removed* from such atmosphere. Again, supposing this to have been a case of yellow fever, there was ample time for the system to "become adjusted to the influence of the poison." Indeed, this was so ample as of itself to make it rather improbable that the patient had the disease at all.

If not yellow fever, what could this disease have been? It may not be easy to determine this. and assign the proper name. Isolated cases frequently occur which defy nosology. Could it have been the effect of bilious remittent fever? This the patient said he had at Kingston, We shall show that black vomit sometimes occurs in this disease, and that the post-mortem lesions, as a whole, agree better with the pathology of bilious remittent than they do with that of yellow fever. Black vomit occurred in the bilious remittent fever in New York in 1843 in the Hospital, and at Yonkers (*Forrey on Rondout Fever*, *N. Y. Jour. of Med.*, vol. i. p. 340). Dr. Dickson (authority none will question) in 1825 saw two patients die of bilious remittent fever, on Charleston Neck, who ejected black vomit from the stomach and bowels. He also saw cases in 1827 (*Essays*, p. 355). Dr. Fenner, of New Orleans, saw two cases of it in 1850 (*South. Med. Reports*, ii. 89). Both these authors, let it be remembered, were familiar with yellow fever. Cleghorn saw black vomit in the tertian fever of Minorca, a disease more unlike yellow fever than bilious remittent is. In the Batavian fever, which was a bilious remittent. Dr. J. Johnson said patients sometimes have black vomit, and occasionally after lingering twenty or thirty days. This occurred also, he says, in the Bengal remittent. Trousseau, Lancisi, Garnier, Imray, and others, refer to the occasional occurrence of black vomit in bilious remittent fevers. Dr. La Roche "has seen an interesting case of copious ejection of well marked black vomit occurring in a fatal attack of colic. It will be recollected in this connexion that Dr. C.'s patient *had great pains in his bowels,* and not in his back, loins, and limbs, as in yellow fever cases. Enough has been said to show that black vomit may occur in bilious remittent fever, even a month after the first attack.

Some of the post-mortem appearances in the case under discussion are more consonant with bilious remittent fever than with yellow fever. The spleen was very soft and friable; Dr. Spier says it was "diffluent." Dr. La Roche remarks that, while the spleen is but little if at all changed in yellow fever, in bilious remittent "it is very generally much enlarged and *softened.*" Dr. Spier says the liver was very dark, between a purple and a chocolate color. This description agrees tolerably well with the so-called "*bronze liver*" of bilious remittent fever. This color, Dr. Alonzo Clark says, is owing to altered *hæmatine,* which Prof. Virchow named *hœmatoidin.* This coloring matter is not in the cells, but in the hepatic tissues. Dr. Spier's drawing well illustrates this. He found the liver well supplied with hæmatoidin. Profs. Clark, Leidy, and Dr. La Roche say, that, though this modified hæmatin is found in black vomit, is has not been *found in the liver of the yellow fever.*

This liver agrees with that of bilious remittent fever in another point. We have already seen that it was but little, if at all, fatty. Prof. Clark, in his letter to Dr. La Roche on the "Brone Liver." says, a deposit of oil globules in the cells and tissues of the liver is *not so frequently met with, nor is it so abundant in remittent as in yellow fever.* In this letter he also makes an interesting remark on the duration of the coloring matter in the bronze liver. He said, "he had examined the livers of two persons who had had remittent fever a year or more before their fatal sickness. In both the remittent color remained well marked, though less intense than in recent cases. The microscope disclosed the coloring matter unchanged, except perhaps, in quantity.

Dr. Speir found the hæmatoidin not only abundant in the liver, but also in many other organs and tissues, This shows how profoundly the blood was modified, The minute and thorough examination made by Dr. Speir, without reference to any theory, has done much towards clearing up the difficulties of diagnosis in this interesting case.

From a careful view of all the facts before us, the most plausible theory of the process of the disease appears to be this. That the patient had, as he alleged, at Kingston, bilious remittent fever which confined him to the hospital several weeks—that for two weeks after leaving the hospital he was imperfectly nourished, his diet being *bread and salt fish;* he did not regain his strength. That although he worked his passage to Philadelphia he was all the while very feeble. Arriving here he dined on corned-beef and cabbage; he vomited in the evening. Next day he took salts and cream of tartar, which caused vomiting and purging; in the evening the ejected matters were copious, acid, and dark-colored. This continued at intervals for thirty-eight hours, when he died. It is probable that the *bilious remittent* fever caused the morbid changes in the blood and in the tissues from which Nature was feebly trying to rally—that the last fatal illness was excited in his enfeebled and altered organism by the ingesta which he took, and by the medicine which was given him.

Dr. Bell, in reply, added, that he thought no one could justly infer from his previous remarks that black vomit necessarily indicated yellow fever, or that it alone was sufficient to stamp a case of disease as such. In addition to what he had said of the symptoms wanting in this case—headache, backache, aching of the extremities, etc.—these, like jaundice, are not unfrequently absent in rapidly fatal cases, in relapse, or in cases enfeebled by other recent disease. There were several such cases among the invalid soldiers under his care last summer. One, in particular, had been sent from the Tortugas on account of dysentery, and at the time he was received into the Floating Hospital his only complaint was an increasing debility. He gradually sank, and died with black vomit in eighteen hours, without other prominent symptoms. There was no question in this case, because the steamer *Delaware*, in which he arrived, was known to be badly infected. Another case also, an invalid from *intermittent fever*, died in twelve hours from the time of attack, with six hours of black vomit. Both of these cases had fatty degeneration of the liver; in one the liver was yellow, in the other it was livid. Besides such cases as these, there is a class of yellow fever patients known as walking cases, which generally die with black vomit within a few hours from the time they come under observaion, and are not usually characterized by chill, headache, or other prominent symptom. Yet they are accepted as true cases of yellow fever.

The case under discussion appears to have been rapidly fatal on account of relapse or other previous cause of debility. It may have been bilious remittent fever, though we have no evidence of this, other than that the patient himself stated that he had that disease at the time when, and the place where yellow fever is known to have been prevailing. The period of incubation is no index of the duration of the disease, nor of its severity, while it is nevertheless highly probable that the fatal issue in this case was promoted by the change from the high temperature where it was contracted to the low temperature under which the position became active.

That a certain period of incubation, chill, pain in head, back, and extremities, jaundice, suffused and injected eyes, and *absence* of thirst—"that *all* of these symptoms are *generally* present in yellow fever"—is contrary to both his observation and his reading; and further, that according to a somewhat extended observation of bilious remittent fever in hot climates, he believes most of these symptoms to be quite as essential to "bilious remittent fever" as to yellow fever. But of black vomit as a symptom, whether existing almost alone, as in this case, or in connexion with other symptoms it is certainly much less characteristic of bilious than of yellow fever under any circumstances whatever; and he is therefore wholly unable to see the propriety of taking this symptom to indicate a disease in which it very rarely occurs, rather than one in which it very commonly occurs.

In addition to what has been said of the pathology of the case, it is certainly quite as reasonable to find "a few cells in process of fatty degeneration," and to infer, therefore, that this lesion has but just commenced, as it is not to be satisfied with anything less than the degree of fatty degeneration common to the drunkard's liver in hospital practice.

The presence or absence of hæmatoidin may depend upon the degree of disorganization of the blood corpuscles. This is a recent question in yellow fever, and requires further investigation. The other questions of Dr. Enos are abundantly answered by the authority quoted—Dr. La Roche.

### *Umbilical Hemorrhage.*

Dr. Conkling reported a case of umbilical hemorrhage:

The hemorrhage first took place on the third day after the birth of the child, and seemed to exude first at the junction of the substance of the cord with the skin, and was very profuse. After applying *pressure* and the *solution* of the *persulphate of iron* without effect, the cord was cut off at its junction with the skin, for the purpose of ligating the vessels, but this operation proving impracticable, *hot needles* were freely used, and with some benefit; but the hemorrhage still continued, until finally the *actual cautery* was applied with success. For six hours after the first application of the *actual cautery*, blood continued to ooze, and the child lost, in this time, about a dram of blood. The cautery was again applied, and was followed by a total cessation of the hemorrhage. Three days afterwards the eschar separated, leaving a healthy surface, and the child is now, at the end of nineteen days, to all appearances, well.

### *Cerebro-Spinal Meningitis.*

DR. CONKLING also reported a case of *cerebro-spinal meningitis:*

On the 7th of February he was called to see J. V., aged 62, who has been suffering with severe supra-orbital pain for several weeks, and for the last three days had been confined to his bed. The day after the doctor first saw him, he had a convulsion which lasted about five minutes. He soon afterwards rallied, and for the subsequent nine days he was well enough to walk the room. On the 17th of February he had another and more severe convulsion. Two hours afterwards he was roused to consciousness, but could not articulate. He had partial paralysis of the right side. He lay in a state of partial stupor during the next day, but frequently complaining of pain on the left side of the head, which he pointed out by describing with his finger a small circle on the part complained of. He died on the morning on the 24th of February.

The post-mortem, made by Dr. Speir, revealed a thickening of the meninges, with infiltration of serum and pus into the sub-arachnoid tissue over the entire surface of both hemispheres, and of the cerebellum. In the anterior part of the left hemisphere was an abscess, an inch in diameter containing ℥ ss. of thick, greenish pus. The abscess communicated, by a small opening, with the left lateral ventricle, and was lined by a vascular membrane, The ventricles of the brain were filled with sero-purulent effusion, and their lining membranes greatly congested. Under the microscope the cerebral substance was found to be softened and to consist of *disintegrated nerve tubes*, *granular cells*, and molecules.

DR. OTTERSON called the special attention of the Society to the importance of the treatment in the case of *umbilical hemorrhage*, and to the unusual result in this case, nearly all such cases proving fatal.

DR. BURGE had successfully used the *persulphate of iron* in a case of umbilical hemorrhage, and thought it, perhaps, the best remedy.

DR. CONKLING stated that of seventy-nine cases, reported in the *New York Journal*, there were but nine recoveries, one by the *thimble* and one by the *nitrate of silver*.

